# Reliability and efficiency of corneal thickness measurements using sterile donor tomography in the eye bank

**DOI:** 10.1007/s10561-021-09980-2

**Published:** 2021-11-13

**Authors:** Loïc Hamon, Adrien Quintin, Stephanie Mäurer, Isabel Weinstein, Achim Langenbucher, Berthold Seitz, Loay Daas

**Affiliations:** 1 Department of Ophthalmology, Saarland University Medical Center (UKS), Kirrberger Straße 100, Bldg. 22, 66421 Homburg/Saar, Germany; 2 Institute of Experimental Ophthalmology, Saarland University, Homburg/Saar, Germany

**Keywords:** Donor cornea, Eye bank, Reliability, Efficiency, Optical coherence tomography, Organ culture

## Abstract

To evaluate the reliability and efficiency of sterile pachymetric measurements of donor corneas based on tomographic data using two different methods: a “manual” and a “(semi-)automated” method. Twenty-five (25) donor corneas (50%) stored in MI and 25 (50%) in MII were imaged 5 times consecutively using an anterior segment OCT (AS-OCT). The central corneal thickness (CCT) was measured both with the manual measurement tool of the AS-OCT (= CCTm) and with a MATLAB self-programmed software allowing (semi-)automated analysis (= CCTa). We analyzed the reliability of CCTm and CCTa using Cronbach´s alpha (α) and Wilcoxon signed-Rank Test. Concerning CCTm, 68 measurements (54.4%) in MI and 46 (36.8%) in MII presented distortions in the imaged 3D-volumes and were discarded. Concerning CCTa, 5 (4%) in MI and 1 (0.8%) in MII were not analyzable. The mean (± SD) CCTm was 1129 ± 6.8 in MI and 820 ± 5.1 µm in MII. The mean CCTa was 1149 ± 2.7 and 811 ± 2.4 µm, respectively. Both methods showed a high reliability with a Cronbach´s α for CCTm of 1.0 (MI/MII) and for CCTa of 0.99 (MI) and 1.0 (MII). Nevertheless, the mean SD of the 5 measurements was significantly higher for CCTm compared to CCTa in MI (*p* = 0.03), but not in MII (*p* = 0.92). Sterile donor tomography proves to be highly reliable for assessment of CCT with both methods. However, due to frequent distortions regarding the manual method, the (semi-)automated method is more efficient and should be preferred.

## Introduction

Since the first keratoplasty was performed by Zirm in 1901 (Zirm 1906), the number of keratoplasties has increased over time (EEBA 2020). According to the German keratoplasty register, 9173 keratoplasties were performed in 2019 in Germany (Flockerzi et al. 2018). The surgical technique has continuously been improved and adapted over the years in order to respond to new challenges. Those improvements of the “original” penetrating keratoplasty (Seitz et al. 2016) included new suture techniques e.g. the double running cross-stitch suture according to Hoffmann (Hoffmann 1976; Suffo et al. 2021), new trephination techniques, e.g. with excimer laser (Seitz et al. 1999), and new lamellar techniques, e.g. the Descemet Membrane Endothelial Keratoplasty (DMEK) (Melles 2006; Seitz et al. 2020) or the Deep Anterior Lamellar Keratoplasty (DALK) (Luengo-Gimeno et al. 2011), optionally assisted with excimer laser (Daas et al. 2021). Other improvements related to corneal grafting are much less recognized or discussed in ophthalmology, such as modern imaging techniques in eye banking, where—in Europe—the donor corneoscleral discs (CD) are prepared and preserved, mostly using organ culture (EEBA 2020). Since the introduction of organ culture in eye banks in 1973 (Summerlin et al. 1973; Doughman et al. 1976), many innovations have been made in terms of improving storage and selection of CD before keratoplasty (Batista et al. 2018). One milestone is the sterile donor tomography which allows easy and sterile tomographic analysis of the CD for clinical decision support in the eye bank before keratoplasty. The possibility of qualifying CD in eye banks using topo- or tomographs had already been considered since 1999 (Terry et al. 1999) but has made significant progress in recent years. Nowadays, donor tomography can be used in clinical routine as a screening method prior to keratoplasty (Seitz et al. 2021; Quintin et al. 2021a) and has already been used, amongst others, to tailor out the best timepoint for changing from MI to MII before penetrating keratoplasty (Wolf et al. 2009; Hamon et al. 2021) to improve the IOL power calculation in classical triple procedure (Quintin et al. 2021b) and to “harmonize” the donor and recipient tomography (Mäurer et al. 2020). However, the corneal tomographs were not developed for this purpose and other challenges when measuring CD under sterile conditions are the optical distortions of the culture container and image deterioration with culture media.

The *purpose* of this study was to investigate the reliability and efficiency (time and resources required for imaging and analyzing the cornea) of the “manual” and “(semi-)automated” measurement of donor central corneal thickness (CCT) through the plastic cell culture flask and through preservative organ culture medium I (MI) or transport/deswelling organ culture medium II (MII) using sterile non-contact donor tomography in the eye bank.

## Materials and methods

No ethical approval was required according to the Ethics Committee of Saarland, Germany. The study was conducted in accordance with the Declaration of Helsinki and surrogate consent for the scientific use of donated corneas was obtained.

In this study, 50 corneas from 40 donors were cultivated in MI (AL.CHI.MI.A. srl, Ponte San Nicolò, Italia) an isotonic medium (307 mOsmol/kg), containing 10% Minimum Essential Medium (MEM), antibiotics (1% Penicillin/streptomycin and 1% Amphotericin B), 1% L-Glutamin, 1.25% Hepes puffer, 3% NaHCO3 and 2% fetal calf serum. Twenty-five (25) (50.0%) corneas were transferred from MI into MII (AL.CHI.MI.A. srl., Ponte San Nicolò, Italia), a hypertonic medium (353 mOsmol/kg), which contains dextran T500 6% in addition to the ingredients of MI. The other 25 (50.0%) corneas remained in MI. All CD were stored at 34 degrees Celsius (°C) in a plastic cell culture flask (Primaria 25 cm^2^ canted-neck cell culture flask, Corning Inc., Corning, NY, USA) containing MI or MII.

A “compromise analysis” was performed with the Software G*Power (Erdfelder, Faul, Buchner, HHU Düsseldorf, Germany) in order to assess the power (1−ß) and the probability of first order error (α) of the statistical analysis for a total sample of 50 corneas (Faul et al. 2009). Statistical analysis were performed using a Wilcoxon signed-rank test; a power (1−ß) of 0.94 and a probability of error (α) of 0.06 were assessed, with effect size (d) according to Cohen of 0.5 (medium effect size) (correspond to 0.5 pooled SD between the means of both groups).

**Fig. 1:**
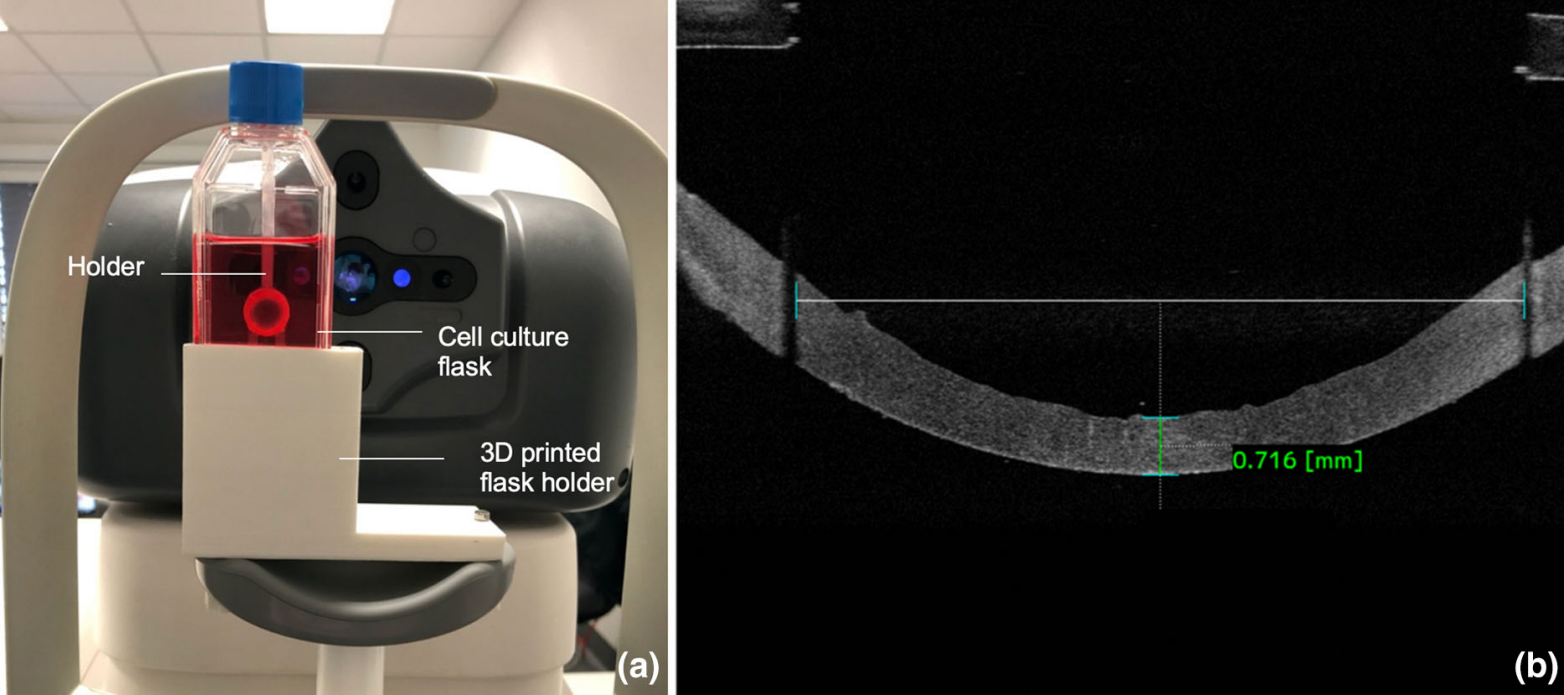
Measurement and determination of the central corneal thickness using the AS-OCT software (“sterile donor tomography”). **a** The measurements were performed under sterile conditions through a cell culture flask using the anterior segment optical coherence tomograph (AS-OCT). The cell culture flask was positioned in a holder adapted to the chin rest of the AS-OCT. **b** The central corneal thickness (CCTm) was manually measured at the bisection between the shaded artefacts caused by the holder

The CD were imaged with an anterior segment optical coherence tomograph (AS-OCT) CASIA 2 (Tomey, Nagoya, Japan) according to the method developed by Mäurer, Damian and Langenbucher (Janunts et al. 2016; Damian et al. 2017; Mäurer et al. 2019) under sterile conditions through their cell culture flask (Primaria 25 cm^2^ canted-neck cell culture flask, Corning Inc., Corning, NY, USA). The culture flasks were maintained on a 3D-printed plastic holder adapted to the chin rest of the AS-OCT (Fig. 1a). The OCT system was aligned to measure the CD from the endothelial side, whereby a central measuring range of approximately 7 mm is achieved, limited by the tissue holder. Out of the initial raster scan of 12 × 12 mm (64 slices, 4 × repeat), subsequent 3-dimensional (3D) volume data were generated with an axial/lateral resolution of 5.6/6 μm/voxel, respectively (Janunts et al. 2016). Each CD was imaged 5 times consecutively by the same examiner (ophthalmologist working in the eye bank and trained to perform the sterile imaging) in MI or MII.

The central corneal thickness (CCT) was assessed using two alternative methods. Firstly, using the manual measurement tool of the AS-OCT (CASIA 2 Software, version 3G.3, Tomey, Nagoya, Japan) with a modified method inspired by that of Schnitzler et al. (2016). The “manually measured” CCT (CCTm) was determined by the corneal thickness at the vertical bisection of the horizontal line between both shaded artefacts caused by the holder (Fig. 1b). All CCTm were measured by the same examiner: a physician specialized in eye banking and trained for “sterile donor tomography”. Secondly, the (semi-)automated CCT (CCTa) was assessed with a MATLAB (The MathWorks Inc., Natick, Massachusetts, USA) custom-made program developed by Mäurer et al. (2019). This software was validated using a polymethyl methacrylate (PMMA) phantom cornea based on Gullstrand’s proportions. While processing the raw data from the AS-OCT, the program eliminates artefacts such as the cell culture flask wall and cornea holder and fits the spherocylindrical model to the corneal front and back surface after correction of image distortion. The corneal thickness is extracted from the model data of both corneal surfaces (Mäurer et al. 2019).

To investigate the reliability of the measurements, Cronbach’s alpha (α) was calculated for the sequence of 5 measurements, separately for each culture medium (MI and MII) and for each pachymetry method (CCTm and CCTa) (Cronbach 1951). For clinical purposes, a Cronbach’s alpha exceeding 0.9 indicates proper reliability (Nunnally et al. 1993).

We calculated the mean of standard deviations (SD) of the 5 subsequent CCTm and CCTa measurements to determine the repeatability. Differences in inter-method agreements were calculated using linear regressions and a Wilcoxon signed-rank test. The results were presented in Bland–Altman plots.

In addition, we analyzed the effect of the storage time in MI or MII (Time in medium—TiM) on the CCT using Pearson’s correlation tests.

Statistical analysis was performed with SPSS Version 20.0.0 for Windows (SPSS Inc., Chicago, IL, USA). Values are expressed as mean ± SD (minimum – maximum).

## Results

**Fig. 2:**
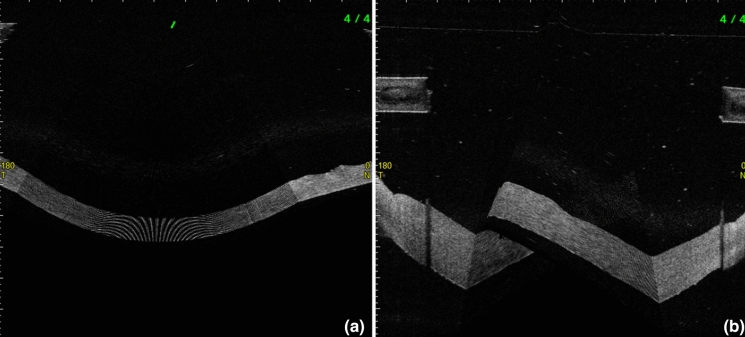
Distortions in the corneal volumes generated by the AS-OCT. Distortions of the 3D corneal volume, visible during manual measurement on the CASIA 2 Software Version 3G.3, presumably due to light reflections on the plastic surface (**a**) and/or to (micro-)movement of the cornea during the raster scan (**b**). These images do not show a sufficient quality for a postprocessing of the 3D volume data and an extraction of CCT

The CCTm was 1129 ± 6.8 µm (710–1923 µm) in MI and 820 ± 5.1 µm (621–1493 µm) in MII. Out of the total of 125 AS-OCT images in each medium, 68 (54.4%) “manual” measurements in MI and 46 (36.8%) in MII had to be discarded due to distortions in the corneal volume generated by the AS-OCT Software (Fig. 2). None of the 5 images were even analyzable for 2 corneas (4%) for MI and 1 cornea (2%) for MII. The CCTa was 1149 ± 2.6 µm (730–1705 µm) in MI and 811 ± 4.4 µm (634–1057 µm) in MII. For 5 cases in MI (4%) and 1 case in MII (0.8%), the MATLAB program could not extract the edges from the volume data and, therefore, CCTa was not available. All individual measurements are shown in Tables 1 and 2.

**Table 1:** Manual and (semi-)automated measurements of central corneal thickness (CCT) in organ culture medium I (MI) Empty fields correspond to images that were not measurable (due to distortions in the 3D volumes—see Fig. 2)

Cornea	Corneal thickness in medium I													
	CCTa [µm]							CCTm [µm]						
	Measure 1	Measure 2	Measure 3	Measure 4	Measure 5	M	SD	Measure 1	Measure 2	Measure 3	Measure 4	Measure 5	M	SD
1	1190	1189	1205	1205	1201	1198	7.0			1234			1234	
2	1191	1211	1209	1211	1212	1207	8.1			1317			1317	
3		1313	1324	1312	1320	1317	5.2	1267	1267	1277	1274	1254	1268	7.9
4	817	815	816	818	816	816	1.0	831	796		821	821	817	12.9
5	1385	1384	1383	1386	1392	1386	3.1				1425		1425	
6	1240	1239	1239	1239	1239	1239	0.5		1247	1199	1172	1222	1210	27.7
7	886	902	883	881	884	887	7.7	761		751			756	5.0
8	1149	1152	1153	1150	1147	1150	2.0							
9	1384	1381	1383		1384	1383	1.2		1352				1352	
10	1074	1075	1074	1076	1075	1075	0.7					984	984	
11	985	985	985	985	985	985	0.2		1044		1014	1021	1026	12.8
12	935	933	926	937	936	933	4.0				834	836	835	1.0
13	1205	1215	1219	1217	1218	1215	5.0					1292	1292	
14	734		731	730	731	731	1.5	710	716				713	3.0
15	1160	1147	1169	1170	1165	1162	8.5	1089	1086	1081		1071	1082	6.8
16	1028	1027	1030	1030	1029	1029	1.1	1014	1006	1006			1009	3.8
17	1234	1258	1254	1251	1255	1250	8.4	1237				1224	1231	6.5
18	921	923	929	922	925	924	2.8	941	974	951		961	957	12.2
19	1077	1069	1077	1076	1078	1075	3.4			1029		1026	1028	1.5
20	1371	1362	1360	1367		1365	4.2	1397			1409	1412	1406	6.5
21	1702	1705	1704	1697	1702	1702	3.0	1905	1913	1923	1910	1913	1913	5.9
22	1217	1214	1214	1219	1214	1216	2.0	1247	1247	1262			1252	7.1
23	1439	1441	1447		1443	1442	2.9					1359	1359	
24	1318	1324	1318	1319	1321	1320	2.4							
25	1214	1213	1217	1206	1219	1214	4.4		1217		1124		1171	46.5
Mean						1149	2.6						1129	6.8

CCTm, manual measure of central corneal thickness; CCTa, (semi-)automated measure of central corneal thickness; M, mean; SD, standard deviation

**Table 2:** Manual and (semi-)automated measurements of central corneal thickness (CCT) in organ culture medium II (MII) Empty fields correspond to images that were not measurable (due to distortions in the 3D volumes—see Fig. 2)

Cornea	Corneal thickness in medium II													
	CCTa [µm]							CCTm [µm]						
	Measure 1	Measure 2	Measure 3	Measure 4	Measure 5	M	SD	Measure 1	Measure 2	Measure 3	Measure 4	Measure 5	M	SD
1	886	886	890	888	886	887	1.9	924	931	924	924	916	924	4.7
2	900	889	890	889		892	4.4	889	894	886	894	881	889	4.9
3	786	783	782	783	785	784	1.4	811	806	804	806	811	808	2.8
4	800	799	799	799	797	799	1.9		776	771	781	770	775	4.3
5	867	866	867	866	869	867	1.2				1149		1149	
6	825	825	825	823	824	825	0.8		871				871	
7	1023	1023	1027	1023	1026	1024	1.7		914		919	929	921	6.2
8	634	635	638	635	635	635	1.2		626				626	
9	721	733	733	734	734	731	5.1		701			708	705	3.5
10	909	911	909	913	911	911	1.7							
11	887	890	889	887	890	889	1.3	941		931			936	5.0
12	685	685	684	683	683	684	0.9	698	706	696	701	698	700	3.4
13	706	705	707	707	705	706	0.8	708	708	691	686	698	698	8.8
14	786	788	803	787	790	791	6.4				757		757	
15	710	724	736	736	733	728	9.9	769	774				772	2.5
16	703	697	699	699	698	699	1.9	681	681	682	686	671	680	4.9
17	918	918	916	913	913	915	2.3	921	911	909	914	919	915	4.6
18	726	721	689	689	726	710	17.6	623	623	621	621	621	622	1.0
19	819	815	826	816	826	820	4.9			791		784	788	3.5
20	1057	1043	1053	1055	1045	1051	5.6	1071	1493				1282	211.0
21	894	890	887	889	897	891	3.6	951	951	961	951	956	954	4.0
22	768	765	768	768	768	767	1.2	789	796	791		794	793	2.7
23	778	775	776	775	775	776	1.0	744		751	774		756	12.8
24	723	726	728	725	727	726	1.9	736	724		726		729	5.2
25	931	951	948	930	949	942	9.4	949				941	945	4.0
Mean						811	2.3						820	5.1

CCTm, manual measure of central corneal thickness; CCTa, (semi-)automated measure of central corneal thickness; M, mean, SD002C standard deviation

Based on available measurements, Cronbach´s α showed a very high reliability for CCTm in MI and MII with a value of α = 1.0. The CCTa showed a very high reliability in MI (α = 1.0) and in MII (α = 0.99).

**Fig. 3:**
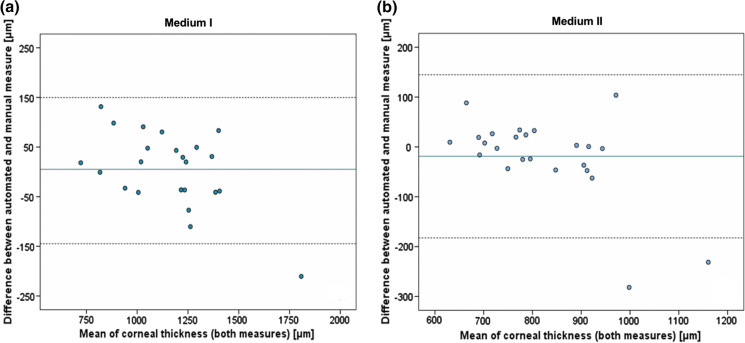
Bland–Altman plots comparing CCTa and CCTm. Limits of agreement regarding mean values of CCT in MI (**a**) and MII (**b**) comparing CCTa and CCTm. Mean value (continuous line) and ± 1.96-fold standard deviation (dotted lines) are illustrated. CCT, central corneal thickness; CCTm, manual measurement of CCT; CCTa, (semi-)automated measurement of CCT; MI, organ culture medium I; MII, organ culture medium II

The level of agreement between CCTm and CCTa measurements were compared using linear regression and Bland–Altman plots (Fig. 3**)**. In both culture media (MI and MII), CCTa and CCTm did not differ significantly for MI (mean difference between the measurements of 5 ± 76 µm, significant level of agreement, *p* = 0.006) and for MII (mean difference between the measurements of 18 ± 83 µm, significant level of agreement, *p* = 0.002). A Wilcoxon signed-rank test comparing the mean SD of both methods showed no significant difference for CD imaged in MI (respectively 6.8 µm and 2.6 µm, *p* = 0.09), but showed a significant larger value for SD with CCTm (5.1 µm) in comparison to SD with CCTa (2.4 µm) (*p* = 0.03) in MII, indicating a better repeatability of measures conducted (semi-)automatically in comparison to manually in MII.

**Fig. 4:**
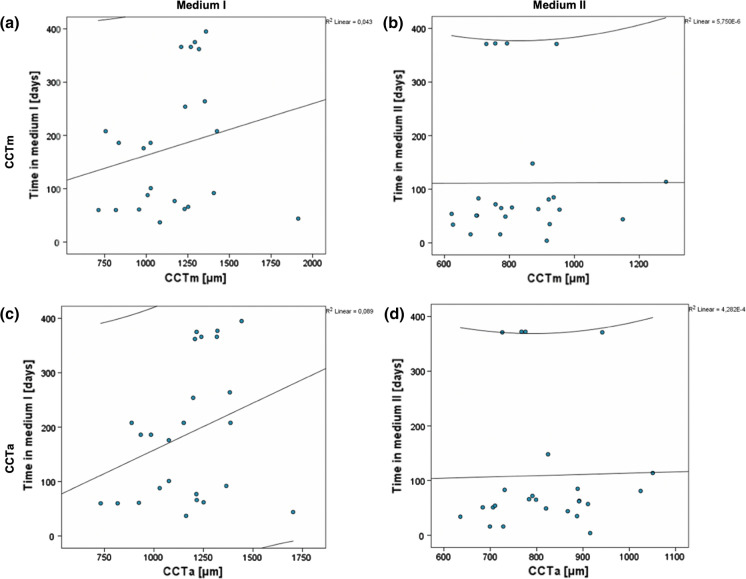
Correlation between TiM and CCT. Pearson’s correlation test showed no correlation between TiM-MI and CCTm (*p* = 0.34) (**a**), between TiM-MI and CCTa (*p* = 0.15) (**b**), between TiM-MII and CCTm (*p* = 0.46) (**c**) and between TiM-MII and CCTa (*p* = 0.92) (**d**). MI, organ culture medium I; MII, organ culture medium I; CCT, central corneal thickness; CCTm, manual measurement of CCT; CCTa, (semi-)automated measurement of CCT; TiM, time during which the sclerocorneal disc (CD) remained in the medium, TiM-MI: TiM in MI, TiM-MII: TiM in MII

The CD storage time in medium I (TiM-MI) or medium II (TiM-MII) did not correlate significantly with CCTm (*p* = 0.34 and *p* = 0.46, respectively) (Fig. 4a, b) or with CCTa (*p* = 0.15 and *p* = 0.92, respectively) (Fig. 4c, d).

## Discussion

In the last few years, new techniques have been developed to improve the quality of corneas in the eye bank. Anterior segment optical coherence tomographs (AS-OCT) represent an interesting option for the analysis of corneoscleral discs (CD) in sterile conditions, including pachymetry and evaluation of front and back surface curvature (Seitz et al. 2021). However, existing AS-OCT instruments are not developed for such applications of *ex-vivo* imaging.

Numerous studies have measured corneal thickness of donor corneas in the eye bank using (AS-)OCT. Neubauer et al. measured the CD through their plastic flask and MII with an OCT Zeiss (model unknow), software version A5 (Carl Zeiss Meditec, Dublin, CA, USA) (Neubauer et al. 2002). Brown et al. measured residual CD after lamellar keratoplasties in MII or glutaraldehyde using a custom ultra-high-resolution OCT (Brown et al. 2008). Wolf et al. measured the donor corneas in whole globes which was disposed on a holder placed on a metal plate (before preparation of the CD) with a Zeiss OCT, software version A5 (Carl Zeiss Meditec, Dublin, CA, USA). They validated the method after comparing the measurements of 16 hydrated corneas using the AS-OCT vs. ultrasound (Wolf et al. 2009). Amato et al. measured posterior donor lenticle (without the anterior cornea lamella) with a Zeiss Visante AS-OCT (Carl Zeiss Meditec, Dublin, CA, USA). They also validated the measurements comparing them to ultrasound and found a very high reliability and repeatability for CCT (Amato et al. 2011). Schnitzler et al. measured the CD through Böhnke organ culture flasks and MII with a Spectralis AS-OCT (Heidelberg Engineering, Heidelberg, Germany) (Schnitzler et al. 2016). Golla et al. measured the CD stored at 4 °C through Transend chamber and Life 4 °C media (Numedis, Isanti, MN, USA) using a Fourier-domain AS-OCT RTVue (Optovue, Fremont, CA, USA) (Golla et al. 2018). Al Bourgol et al. measured the donor corneas in a Petri dish (without liquid) with an AS-OCT CASIA SS-1000 (Tomey, Nagoya, Japan) (Al Bourgol et al. 2021).

The disparity of OCT devices (using different optical sources and spatial resolutions (Ang et al. 2018)) and the different measurement conditions do not allow a direct comparison of the results presented in the studies mentioned above. None of these studies reported the use of an optical correction for image distortion in addition to those presumably already applied by the AS-OCT systems themselves. Wolf et al. as well as Amato et al., showed—as mentioned above—tomographic CCT values as being similar to ultrasonic CCT measurements (performed for model validation). However, these measurements were performed with an old OCT model on whole globes and on posterior donor lenticle, respectively*.* These results have therefore a low clinical relevance for current CD measurements.

In this study, we compared the results of two types of corneal measurements of the raw data obtained with the AS-OCT CASIA 2 (Tomey, Nagoya, Japan), a Swept Source (SS) AS-OCT, using a raster scan, through a plastic flask filled with MI or MII. The “manual” measurements (CCTm) were performed with the CASIA 2 Software version 3G.3, using the raw 3D volume data imaged and post-processed by the AS-OCT system. The instrument manufacturer does not provide information about spatial and optical built-in corrections applied in the images themselves, and we did not apply any additional correction factor after the measurements. The (semi-)automated measurements (CCTa) were perform by the customized MATLAB software, according to Mäurer, Damian, Langenbucher et al. (Janunts et al. 2016; Damian et al. 2017; Mäurer et al. 2019), that processed the same raw 3D volume data imaged with the AS-OCT. The software fits a spherocylindrical model to both corneal surfaces based on the 3D volume data after removing artefacts from the cell culture flask and the medium, and corrects the spatial (geometric) and optical (refractive) distortions that may be caused by the axial scan through the plastic flask and the organ culture medium. This method was validated using a corneal-like PMMA phantom with design data according to the Gullstrand schematic model eye (Damian et al. 2017; Mäurer et al. 2019). The two methods showed very similar CCT results, with CCTm = 1129 ± 6.8 µm versus CCTa = 1149 ± 2.6 µm in MI and CCTm = 820 ± 5.1 µm versus CCTa = 811 ± 4.4 µm in MII, which were comparable (*p* = 0.006 in MI; *p* = 0.002 in MII). As the CCTa values have previously been validated, these results suggest that CCTm values do not require additional correction and could be used as generated by the AS-OCT system. This result may seem surprising since even with the plastic cell culture flask and the medium (I or II) on both sides of the CD the tomographer is not working under normal conditions with air/cornea/aqueous humor interfaces.

The results were strongly reliable for both CCTm and CCTa methods in both organ culture media but showed a higher repeatability using the CCTa, not significant in MI (*p* = 0.09), however significant in MII (*p* = 0.03). However, the CCTm was impaired in 54.4% of the cases in MI and 36.8% of the cases in MII due to the presence of distortions in the post-processed volumes of the AS-OCT Software due to artefacts, i.e. light reflections on the plastic surface or micro-movements during the examination (Fig. 2). A solution to this problem may be to perform several successive images to obtain "at least" one analyzable measurement. Nevertheless, we showed that in 6% of the corneas, all 5 images were not sufficient to properly measure the CCTm of one image. According to our experience prior to the implementation of the custom MATLAB software, the number of AS-OCT imaging required to obtain an analyzable 3D model can be up to 10, considerably increasing the time required to measure the CCT for each individual cornea. This might put into question the statements about “repeatability” or “reproducibility”, despite the seemingly high reliability of CCTm (based on measurable 3D-volumes). In contrast, the MATLAB software was able to generate a measurable 3D-volume for 96% of the CD in MI and 99.2% of the CD in MII. Considering the issue caused by the artefacts and the considerable time saving by using the custom MATLAB software (both for imaging and analysis), the semi-automated method seems to be much more efficient to analyze corneal transplants in clinical practice.

As part of this study, we also analyzed the effect of the CD storage time in MI or MII (TiM) on the absolute CCT values (Fig. 4). This analysis showed no effect of TiM on absolute CCT values (neither CCTm nor CCTa). While this seems to apply under stable conditions (prolonged storage in the same medium), there is evidence nonetheless that the cornea swells in MI (Pels 1997; Doughman 1980) and deswells in MII during the first hours after the transfer (Borderie et al. 1997; Hamon et al. 2021).

In practice, an AS-OCT remains an expensive device and the procurement of such equipment for the eye bank is questionable. Being part of a university corneal transplantation center with on-site eye bank, we use the same AS-OCT that is used daily in the framework of our consultations. As a standard procedure, tomographic images of all CD are performed preoperatively at our Department of Ophthalmology since 2018 (Seitz et al. 2021). This option—available in the majority of large ophthalmology centers—does not require any additional costs. For the pachymetrical measurements, we use the (semi-)automated method using our self-programmed MATLAB software, which almost systematically allows us to obtain a CCT value. The generation of a 3D model presents advantages compared to a single central measurement with cross-line scan. Firstly, a 3D model allows a complete mapping with not only a central measurement (CCT) but also peripheral measurements. If the peripheral measurements are generally of minor importance, they may enable the detection of specific abnormalities such as marginal pellucid degeneration or an ulcer, for example. Secondly, this self-programmed MATLAB software contains others functionalities which require 3D modeling, such as a keratometric analysis (steep and flat anterior and posterior radii of curvature) with detection of curvature anomalies (Damian et al. 2017) and an automated detection of corneal opacities (stromal scars, corneal flap after laser in situ keratomileusis (LASIK), corneal dystrophies, …) (Seitz et al. 2021). This information is of great importance for eye banks, considering that a cornea with curvature anomaly or stromal densification can be used—for example—for DMEK, if the endothelial cell count (ECC) is sufficient. All these functionalities run in an integrated and simultaneous sequence after a single imaging, which supports the efficiency of this semi-automated method in comparison to a manual CCT measurement or a single central cross-line scan. In the future, curvature mapping could allow to orient the transplant during the surgery in order to align the steep and flat meridians of donor and recipient together and thus minimize postoperative astigmatism (“harmonization” of donor and recipient tomography) (Mäurer et al. 2021). Until today, this self-programmed MATLAB software was used to analyze raw data from 3DV-files from CASIA or CASIA 2 AS-OCT (Tomey, Nagoya, Japan) and from AVI-files from Spectralis AS-OCT (Heidelberg Engineering, Heidelberg, Germany). However, the software should be able to use 3DV or AVI-files from others models of AS-OCT with little or no specific adjustments. This software is currently not available for sale or download for other departments or eye banks and is still subject to research projects. In the future, it could be made available if there is sufficient demand.

This study has two limitations. First, the fixation of the CD to the cell culture flask holder may cause a slight deformation of the cornea, whereby the measured geometry may not fully correspond with “*in vivo*” conditions. Second, the measurements do not take into account the inhomogeneous epithelial layer, which could cause a bias of approximately 50 µm (Neubauer et al. 2002). Since the condition of the corneal epithelium is assumed to be comparable with sequential measurements of the same CD, this limitation could affect the absolute measurement of CCT, but is not expected to affect measures such as reliability or repeatability.

In conclusion, both manual and (semi-)automated methods using the AS-OCT CASIA 2 showed a high reliability in both culture media I and II. Despite the similarity between manual and (semi-)automated measurements, manual measurements were frequently hampered by artifacts or distortions. For this reason, semi-automated measurements seem to be more efficient and should be preferred.

## Data Availability

Data and material were provided and collected in the Department of Ophthalmology, Saarland University Medical Center (UKS), Homburg/Saar, Germany. Data will be made available on reasonable request.

## Code availability

«Corneal Tomography” is a MATLAB Software programmed by the Institute of Experimental Ophthalmology, Saarland University, Homburg/Saar, Germany. Data will be made available on reasonable request.
